# The Hospital Treatment of Persons Who Have Attempted to Commit Suicide

**Published:** 1913-01-25

**Authors:** 


### PROBLEMS IN ADMINISTRATIVE MEDICINE.

(Carefully thought-out Contributions to this Section will be welcome.)

### The Hospital Treatment of Persons who have Attempted to Commit Suicide.

Anyone who visits hospital wards at all frequently is certain, sooner or later, to observe a uniformed police-constable sitting or standing near the bed of a patient. If such a visitor, surprised at this unexpected sight, inquires of the sister or staff nurse why the constable should be there, in nine cases out of ten he will discover that the patient thus under surveillance is one who has attempted unsuccessfully to commit suicide. In medical wards the method selected will usually have been poisoning or drowning; in surgical, some form of violence, such as cutting the throat, jumping from a window, or shooting. The patient thus admitted is treated in the first place by the house physician or surgeon, as the case may be, on duty at the time; and subsequently by the same individual under the supervision of a member of the medical, or surgical, visiting staff.

When the patient is sufficiently recovered to be no longer detained he is handed over to the police, and may or may not be charged before a legal tribunal for the criminal offence of attempted suicide. As a matter of actual practice, probably only a minority of the offenders are ever thus placed on trial; three out of four, perhaps, are allowed to return to their friends without undergoing that unpleasant ordeal. But, strictly speaking, every such would-be suicide has committed a crime; and it lies within the discretion of the authorities to prosecute if they think fit. That is, of course, the reason for the presence of the constable in the ward. He is there to prevent the possible escape of the malefactor, for the hospital managers cannot be held accountable should he disappear.

### The Patients' Point of View.

In point of fact, the constable in these circumstances often makes himself very useful. A comfortable chair in a warm ward is better fun than point duty on a wet winter's day; and the sister can often get sundry small jobs done in the ward by being tactful in her dealings with the visitor. If the patient should be in need of control, as in delirium tremens or other forms of mania, the assistance of a stalwart constable is no small help to the sometimes decrepit "male special"; and isolation rooms are not always available for such cases. So, too, a depressed melancholic patient may be on the look-out for a chance of completing his hindered design; here, again, physical force may be needed to prevent him from cutting his throat with a table knife, or jumping from the ward window.

What about the patients' point of view? Alas, it is not very often considered. For calling attention to it, and for a very sound practical suggestion in the matter, the thanks of hospital managers are due to the Editor of the *Guy's Hospital Gazette*,* who offers some sane and common-sense comments upon the subject. These patients, he says, and we can corroborate the remark for other leading hospitals besides Guy's, are never considered as they should be from the point of view of the mental disorder from which they are suffering. Medically and surgically their treatment is admirable; but the after-history of a considerable proportion of the patients is lamentable, according to this writer. The suggestion is offered that in every case of attempted suicide admitted to the wards, the diagnosis and treatment of the mental condition should be referred to Dr. Maurice Craig, the alienist to Guy's Hospital and lecturer on psychological medicine to the medical school.

### A Suggested Reform.

Such a routine course of action would be to the advantage of the patient; and also, be it noted, of the students, who would be able to profit by the rare opportunity of first-hand experience in psychological medicine, demonstrated by an expert alienist. Merely to treat the cut throat or the corrosive poisoning or what not is, as the Editor of the *Guy's Hospital Gazette* truly says, treating the symptoms only, and ignoring the disease. He is in favour also of banishing the constable from the patient's bedside, on the ground that his presence may worry the patient and retard his recovery. From the point of view of the other patients there is, however, a good deal to be said in favour of retaining him.

The process of recovery from most forms of suicide—and especially perhaps after attempted poisoning and cut throat—is so excessively unpleasant that most of those who have been through it, apart from the actually insane, are little likely to run the risk of going through it again. The barely adolescent youth or maid who swallows carbolic acid or laudanum because of a temporary estrangement from the object of his or her affection, or for some similar evanescent sense of tragedy, can usually be trusted not to be so silly another time. With this class, not by any means a small one, the alienist would have little trouble. Indeed, the future of such indiscreet persons is often best confided to the hospital chaplain's supervision, or to that of some charitable organisation; and even graver cases of mental instability can sometimes be safely entrusted to similar agencies with advantage. But there are also many more intractable cases for which such after-care is quite insufficient. It would be the duty of the alienist to indicate them, and to recommend such steps as he might deem best for their future control and amelioration. At any rate, the suggested consultation with an alienist over every case of attempted suicide admitted to a hospital is one which deserves very serious consideration from hospital governing bodies, and is, in our opinion, well worthy of adoption.

* December 7, 1912, p. 489.

